# Inflammatory Memory of Adipose Tissue Macrophages: From CD68 Footprint to Cardiometabolic and Cancer Risk During Weight Cycling

**DOI:** 10.3390/ijms27104203

**Published:** 2026-05-08

**Authors:** Dragana Tomić Naglić, Mia Manojlović, Slađana Pejaković, Nikolina Vuković, Teodor Grbić, Ognjen Milanović, Milan Mirković, Slobodan Maričić, Tamara Maksimović, Andrijana Milankov

**Affiliations:** 1 Faculty of Medicine, University of Novi Sad, 21137 Novi Sad, Serbia; dragana.tomic-naglic@mf.uns.ac.rs (D.T.N.); 910060d23@mf.uns.ac.rs (N.V.); milanovicognjen@gmail.com (O.M.); milanmirkovic1@hotmail.com (M.M.); slobodan.maricic@mf.uns.ac.rs (S.M.); tamara.maksimovic@mf.uns.ac.rs (T.M.); andrijana.milankov@mf.uns.ac.rs (A.M.); 2 Clinic for Endocrinology, Diabetes and Metabolic Disorders, University Clinical Center of Vojvodina, 21000 Novi Sad, Serbia; teodor.grbic@gmail.com; 3 Regena Polyclinic, 21101 Novi Sad, Serbia; sladjana.pejakovic@gmail.com; 4 Department for Gynecology, Clinic for Surgical Oncology, Oncology Institute of Vojvodina, 21204 Sremska Kamenica, Serbia

**Keywords:** obesity, meta-inflammation, adipose tissue, macrophages, CD68, weight cycling, PI3K/AKT/mTOR, cardiometabolic risk, cancer progression

## Abstract

Obesity is characterized by chronic low-grade inflammation (meta-inflammation) and metabolic dysregulation. Adipose tissue acts as an immunometabolic organ, with macrophages playing a central role. This review examines inflammatory memory in adipose tissue, focusing on CD68+ macrophages and their role in cardiometabolic and cancer risk during weight cycling. (2) Narrative synthesis of evidence from immunology, obesitology, and oncology, with emphasis on macrophage polarization and signaling pathways. (3) Weight cycling induces persistent immune memory in adipose tissue, characterized by exaggerated macrophage responses upon weight regain. CD68+ macrophages contribute to extracellular matrix remodeling, tumor signaling, and metabolic dysfunction. Key mechanisms include PI3K/AKT/mTOR dysregulation, FOXO1/KLF10 axis impairment, and CREB-mediated transcription. This inflammatory memory promotes atherosclerosis progression, insulin resistance, and increased cancer risk, despite prior weight loss. (4) Macrophage-driven inflammatory memory represents a key mechanistic link between obesity, cardiometabolic disease, and cancer. Targeting meta-inflammation independent of body weight should be integral to future therapies.

## 1. Introduction

Definition: Inflammatory memory in adipose tissue macrophages is defined as a persistent, context-dependent state of functional reprogramming induced by prior inflammatory or metabolic stimuli, which enables an altered and typically amplified response upon subsequent challenges. This state is sustained through integrated epigenetic, metabolic, and signaling adaptations and is shaped by the tissue microenvironment, thereby linking past exposures to future cardiometabolic and oncologic risk.

Inflammation within adipose tissue is a central feature of cardiometabolic disease and has increasingly been implicated in cancer development and progression. Among the key cellular mediators of this process are adipose tissue macrophages (ATMs), which dynamically respond to metabolic stressors such as obesity, insulin resistance, and weight cycling. Traditionally, ATM activation has been viewed as a transient response to environmental cues; however, emerging evidence suggests that these cells can acquire long-lasting functional adaptations that persist beyond the initial stimulus [1].

This concept aligns with the broader framework of innate immune memory, often referred to as trained immunity, in which prior exposure to inflammatory or metabolic signals induces sustained changes in immune cell behavior. While this paradigm has been well described in circulating monocytes and bone marrow progenitors, its manifestation within tissue-resident macrophages—particularly in metabolically active environments such as adipose tissue—remains insufficiently defined. In this context, adipose tissue represents a unique immunometabolic niche where chronic nutrient excess, altered lipid flux, and local hypoxia create conditions that may not only initiate inflammation but also imprint long-term cellular memory [2]. In this review, we propose that adipose tissue macrophages develop a distinct form of inflammatory memory that is shaped by both systemic metabolic inputs and local microenvironmental signals. We define inflammatory memory in ATMs as a persistent, context-dependent state of functional reprogramming induced by prior inflammatory or metabolic stimuli, which enables an altered and often amplified response upon subsequent challenges. Importantly, this concept extends beyond classical trained immunity by incorporating tissue-specific factors, including adipocyte–macrophage crosstalk, extracellular matrix remodeling, and metabolic substrate availability.

To address the current lack of conceptual clarity, we present a unifying mechanistic framework in which inflammatory memory in adipose tissue macrophages is established, maintained, and functionally expressed through three interconnected processes: epigenetic priming, metabolic reprogramming, and microenvironmental reinforcement. Within this framework, prior exposures are encoded at the chromatin level, stabilized through metabolic adaptations, and continuously modulated by the adipose tissue niche, ultimately linking past inflammatory or metabolic insults to future disease risk [3,4].

By integrating these mechanisms, this review aims to move beyond a descriptive overview and provide a structured model that explains how inflammatory memory in adipose tissue macrophages contributes to the progression of cardiometabolic disorders and cancer. Furthermore, we highlight the implications of this concept in clinically relevant contexts such as obesity, weight cycling, and metabolic recovery, where repeated or fluctuating stimuli may reinforce maladaptive immune programming. A clearer understanding of these processes may open new avenues for targeted therapeutic strategies aimed at disrupting or reprogramming inflammatory memory in metabolic disease.

## 2. Three Core Mechanisms of Inflammatory Memory in Adipose Tissue Macrophages

### Epigenetic Priming (Encoding of Prior Exposure)

Definition: Epigenetic remodeling establishes a durable transcriptional bias in macrophages following inflammatory or metabolic stimuli:

- Histone modifications: H3K4me3 (enrichment of this mark at the promoters of pro-inflammatory genes (like TNF or IL6) is a hallmark of trained cells; it keeps these regions in an “open” or “ready” state, even after the initial stimulus is gone).

- Histone modifications: H3K27ac: This acetylation mark serves as a signature for active enhancers. In trained immunity, the deposition of H3K27ac is often mediated by the recruitment of p300, a histone acetyltransferase that physically opens the chromatin to allow transcription factors to bind.

- Chromatin accessibility enhancer priming trained immunity overlap. Trained cells exhibit increased accessibility at thousands of genomic sites. These “latent enhancers” may lack traditional marks in their naive state but gain H3K4me1 and H3K27ac upon stimulation, sometimes retaining these features long-term to facilitate rapid re-activation.

Epigenetic priming encodes prior exposures into a poised transcriptional landscape that facilitates rapid and amplified reactivation [5,6].

## 3. Metabolic Reprogramming (Maintenance of the Memory State)

Definition: Sustained shifts in cellular metabolism stabilize and reinforce the memory phenotype over time. There are not merely consequences of cellular activation, but are fundamental drivers that stabilize, reinforce, and lock in the memory phenotype over time. Metabolic rewiring—often a transition from glycolysis to oxidative metabolism or a specialized hybrid state—acts as a “memory maintenance engine,” where metabolites serve as signals that communicate with the nucleus to lock in epigenetic changes. While rapid inflammatory responses (effector state) rely on aerobic glycolysis for fast ATP production, the formation and maintenance of memory (e.g., T cells, stem cells, neurons) require a metabolic shift towards mitochondrial oxidative phosphorylation (OXPHOS) [7].

Mitochondria in memory cells undergo “rewiring” to change from simple powerhouses into specialized, dynamic organizers of memory. The rewiring includes structural remodeling (mitochondria in memory cells often undergo fusion to form tubular networks, allowing them to better handle energy demands and resist degradation), dynamic signaling (in neurons, spaced training (which forms long-term memory) activates a dopamine-driven signaling pathway), and DAMB-PKCδ, which increases mitochondrial pyruvate consumption and stabilizes the memory and fission/fusion balance (increased mitochondrial fission (via Drp1) in synapses often sustains memory by ensuring that enough small, mobile mitochondria reach the synaptic terminals to fuel synaptic remodeling) [8].

Recent studies indicate that “metabolic memory” extends beyond glucose (hyperglycemia) to include lipid metabolism, where a transient abnormality leads to long-term consequences, often linked to epigenetic modifications that persist after the metabolic state has normalized. Metabolic reprogramming of lipid metabolism is a key mechanism for maintaining the memory state (memory T cells, regulatory T cells) and driving pathological persistence (cancer stem cells, chronic inflammation). In immunological memory, this involves a transition from glycolysis (used by effector cells) to catabolic fatty acid oxidation (FAO). In pathological contexts, such as tumor cells and CAFs (cancer-associated fibroblasts), it involves upregulated lipid uptake (CD36), synthesis (FASN), and storage (lipid droplets), often coupled with epigenetic modifications that persist after the initial metabolic stress. Metabolic rewiring not only fuels inflammatory responses but actively sustains the persistence of the memory state [2,9,10].

## 4. Microenvironmental Reinforcement (Contextual Activation and Reactivation)

Definition: Adipose tissue-specific signals continuously shape and reactivate macrophage memory through local cellular and molecular interactions.

Core Mechanisms of Microenvironmental Reinforcement include:

- Adipocyte-Derived Signals: Adipocytes, particularly when undergoing hypertrophy or stress (as in obesity), secrete signals such as MCP-1 (CCL2) and TNF-α, which recruit and polarize macrophages toward a pro-inflammatory state. Conversely, in lean tissue, adipocytes produce factors promoting M2-like, anti-inflammatory macrophages that maintain tissue homeostasis.

- ECM Remodeling and Stiffness: Obesity-driven rapid adipocyte expansion triggers excessive ECM component deposition (e.g., collagen I, V, VI, elastin, fibronectin). This fibrotic, rigid environment creates a “stiff” microenvironment that directly dictates macrophage activation, often promoting a pro-inflammatory or profibrotic “metabolically activated” phenotype.

- Reactivation via Crown-Like Structures (CLSs): CLSs are clusters of macrophages surrounding dying or necrotic adipocytes. These structures represent a key mechanism for localized reactivation, where CLS-associated macrophages are exposed to high concentrations of metabolic danger signals (lipids, DAMPS), enforcing a persistent “trained” pro-inflammatory memory.

- Macrophage Memory and Trained Immunity: Adipose tissue macrophages (ATMs) can exhibit “innate immune memory,” where prior exposure to factors like saturated fatty acids or adipose-conditioned media increases their reactivity to subsequent inflammatory challenges.

- Bidirectional Crosstalk: Macrophages, in turn, regulate the ECM by secreting MMPs (matrix metalloproteinases) and cytokines like TGF-β1, which can further activate fibroblasts to produce more matrix, creating a self-reinforcing loop of fibrosis and macrophage activation. This continuous feedback loop means that the local adipose environment does not merely activate macrophages once but constantly reprograms their functional state, linking metabolic stress to chronic, localized inflammation.

Obesity/weight cycling is memory trigger in which the adipose tissue microenvironment acts as a dynamic amplifier, determining whether inflammatory memory remains latent or becomes pathologically reactivated.

Together, these three interconnected mechanisms—epigenetic priming, metabolic reprogramming, and microenvironmental reinforcement—form an integrated framework through which inflammatory memory is established, maintained, and functionally expressed in adipose tissue macrophages [11].

The current evidence supporting inflammatory memory in adipose tissue macrophages, including distinctions between human and experimental data, is summarized in Table 1.

## 5. Histopathological Features of White Adipose Tissue Inflammation

The histopathological features of white adipose tissue inflammation are characterized by hypertrophied adipocytes, which exhibit functional impairment and increased susceptibility to cell death. This phenomenon is largely attributable to adipocyte expansion, resulting in an enlarged cellular membrane surface that intensifies interactions with the extracellular matrix and neighboring immune cells. Hypertrophic adipose tissue is also prone to hypoxia, which, together with immune cell infiltration, contributes to the development of dysfunctional adipose tissue [24].

A hallmark histological feature is the presence of macrophages surrounding necrotic or apoptotic adipocytes, forming so-called crown-like structures (CLS). The presence of CLS is not only an indicator of adipose tissue inflammation but also carries prognostic significance. It has been associated with adverse outcomes, including reduced progression-free survival in breast cancer and squamous cell carcinoma of the tongue. Furthermore, CLS formation correlates with key cardiometabolic risk factors, including dyslipidemia, hypertension, hyperglycemia, and established type 2 diabetes mellitus [25].

Additional histopathological features include the accumulation of extracellular matrix components and fibrin deposition, which impair lipid storage capacity within adipocytes and promote ectopic fat deposition [26].

Immunohistochemistry (IHC) plays a crucial role in the evaluation of adipose tissue inflammation. Li et al. demonstrated that increased expression of CD68+ and CD163+ macrophages was significantly associated with poor prognosis in patients with breast cancer (BRCA) [27]. A growing body of evidence indicates that CD68+ macrophages are involved in multiple immune-related biological processes, including adaptive immune responses, macrophage activation, extracellular matrix remodeling, and regulation of DNA metabolism. Moreover, CD68+ macrophages have been linked to tumor-associated signaling pathways, such as the cell adhesion molecule pathway and the JAK–STAT signaling pathway [27,28].

In gastrointestinal malignancies, the prognostic significance of CD68+ macrophages appears to be context-dependent, likely reflecting their interaction with the tumor microenvironment (TME). Despite these inconsistencies, CD68+ macrophages remain a promising potential therapeutic target in selected malignancies. However, further experimental and, importantly, clinical studies are required to clarify their role [28].

The specificity and sensitivity of CD68 as a biomarker are relatively limited when assessed in isolation. Its prognostic value is significantly enhanced when evaluated in conjunction with co-expressed genes and immune markers, particularly those associated with eukaryotic translation initiation factor 4E (eIF4E). Notably, elevated levels of both eIF4E and CD68+ macrophages have been shown to correlate with poor prognosis and increased relapse rates, especially in triple-negative breast cancer [27].

Together, these findings position CD68+ macrophages as a histopathological footprint of adipose tissue inflammatory memory.

## 6. Weight Loss and Regain: Clinical Consequences

Weight loss would be expected to induce remodeling of metabolically active markers of macrophage function, such as CD9, TREM2, LPL, and LIPA; however, this is not consistently observed. On the contrary, as discussed in previous sections, a persistent low-grade macrophage activity often remains even during remission of obesity, representing a primed state that can be rapidly reactivated upon weight regain [28].

This immune cell-mediated memory during weight cycling has significant metabolic and clinical consequences. Experimental studies have demonstrated an acceleration of atherosclerotic processes during weight regain [29]. This observation is further supported by evidence showing that weight regain is accompanied by a rapid increase in macrophage activity, T lymphocyte activation, and pro-inflammatory cytokine production [30].

In parallel, dysfunctional and inflamed adipose tissue contributes to the development of hyperglycemia driven by hyperinsulinemia. Although substantial weight loss can induce long-term remission of type 2 diabetes mellitus, current evidence indicates that metabolic disturbances such as prediabetes and insulin resistance re-emerge more rapidly following weight regain. Notably, during a second phase of obesity, these disturbances tend to be more pronounced and more readily progress toward overt type 2 diabetes [31].

The role of autophagy in pancreatic β-cell function under conditions of impaired glucose tolerance remains incompletely understood. It is unclear whether autophagy exerts a protective effect or contributes to progressive β-cell loss and accelerated diabetes development. Wu et al. reported that extensive autophagy and reduction in β-cell mass occur predominantly under conditions of NR3C1 (glucocorticoid receptor) activation [32]. Histopathological analyses of pancreatic tissue from patients with diabetes mellitus revealed a fourfold increase in autophagic vacuoles without chromatin condensation compared to healthy individuals, consistent with features of autophagy-associated cell death [32]. At the molecular level, pancreatic tissue exhibits significant expression of NR3C1, through which glucocorticoids inhibit insulin secretion as well as β-cell survival and proliferation. Therefore, the effects of autophagy appear to be context-dependent and influenced by NR3C1 expression patterns [33].

It is estimated that approximately 4–9% of all cancer diagnoses are attributable to excess adiposity, and obesity is recognized as a negative prognostic factor for survival in these patients. Epidemiological studies conducted in the early 21st century have demonstrated that obesity significantly increases mortality risk from cancers of the esophagus, colorectum, liver, gallbladder, pancreas, and kidney [34].

Adipose tissue contributes to carcinogenesis by providing an excess of nutrients that support tumor growth. In addition, studies have shown that obesity not only increases PD-1 expression on CD8+ T cells but also selectively upregulates PD-1 on tumor-associated macrophages (TAMs). Obesity-associated factors—including IFN-γ, TNF, leptin, and insulin—promote PD-1 expression via mTORC1 signaling pathways. Subsequently, PD-1 signaling exerts negative feedback on TAMs, suppressing glycolysis, phagocytosis, and antigen-presenting capacity, thereby facilitating tumor progression and impairing anti-tumor immunity [35].

Furthermore, adipose tissue inflammation may contribute to CD8+ T-cell exhaustion by altering the tumor extracellular matrix. Macrophages in close proximity to tumor cells produce TGF-β1, which stimulates collagen production, promotes adipocyte differentiation, and inhibits the thermogenic potential of adipose tissue. This leads to remodeling of the tumor microenvironment into a state that is less permissive for CD8+ T-cell infiltration, thereby significantly reducing the anti-tumor capacity of the immune system [36].

**Figure 1:**
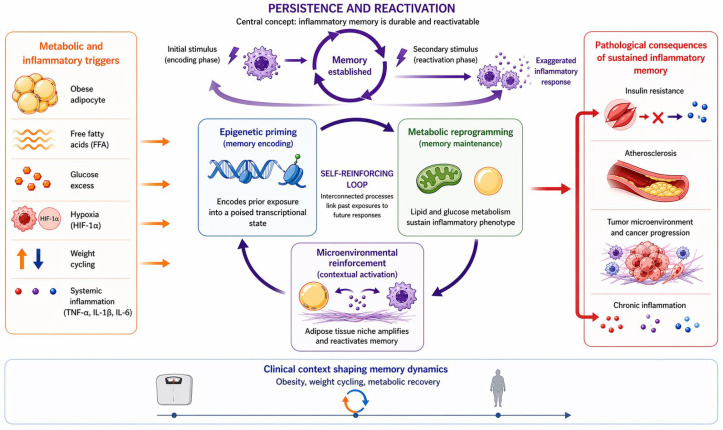
**Proposed model of adipose tissue inflammatory memory during weight cycling.** In the lean state, adipose tissue is characterized by immune homeostasis, with predominance of anti-inflammatory immune cells and cytokines. Weight loss reduces adipose inflammation and macrophage burden, but does not fully erase the immunologic imprint of prior obesity. A persistent CD68-related macrophage footprint may remain within adipose tissue as a form of inflammatory memory. During weight regain, this residual immune programming contributes to exaggerated immune activation, increased M1-like macrophage infiltration, crown-like structure formation, CD8+ T-cell engagement, and reactivation of meta-inflammation. These processes may contribute to increased cardiometabolic and oncologic risk. Colors distinguish the main mechanistic components, arrows indicate the direction of interactions, circular arrows represent self-reinforcing feedback loops, and red arrows/highlighted elements denote pathological consequences and enhanced inflammatory activation.

Together, these findings suggest that weight regain represents not merely a reversal of metabolic benefit, but a biologically amplified state driven by persistent inflammatory memory, as illustrated in Figure 1.

## 7. Therapeutic Perspectives

### 7.1. Targeting Inflammatory Memory in Adipose Tissue Macrophages: A Mechanistic Approach

The concept of inflammatory memory in adipose tissue macrophages (ATMs) provides a framework for rethinking therapeutic strategies in cardiometabolic disease and cancer. Rather than focusing solely on downstream inflammatory mediators, emerging approaches should aim to interfere with the formation, persistence, and reactivation of maladaptive immune programming. Within this context, targeting inflammatory memory can be conceptualized across three interconnected levels: epigenetic priming, metabolic reprogramming, and microenvironmental reinforcement [37,38].

### 7.2. Targeting Memory Formation: Modulation of Epigenetic Priming

The establishment of inflammatory memory is initiated through epigenetic remodeling, which encodes prior inflammatory or metabolic exposures into a persistent transcriptional landscape. Interventions at this stage aim to prevent or attenuate the initial imprinting of maladaptive immune responses. Experimental studies have identified histone-modifying enzymes and chromatin regulators, including histone deacetylases and bromodomain-containing proteins, as potential targets for modulating inflammatory gene accessibility. Although direct clinical application remains limited, early anti-inflammatory interventions and timely metabolic control may indirectly reduce epigenetic priming in adipose tissue. Importantly, the reversibility of these epigenetic marks remains an area of active investigation, raising the possibility that early therapeutic intervention could have long-term benefits by preventing the establishment of persistent inflammatory memory [37,39,40,41].

### 7.3. Targeting Memory Persistence: Metabolic Reprogramming as a Therapeutic Axis

Metabolic reprogramming represents a central mechanism sustaining the inflammatory memory phenotype in ATMs. Persistent alterations in glucose metabolism, mitochondrial function, and lipid handling not only fuel inflammatory responses but also stabilize the memory state over time. This provides a clinically relevant opportunity to disrupt inflammatory memory through metabolic interventions. Agents such as metformin, which modulate mitochondrial activity and improve insulin sensitivity, as well as glucagon-like peptide-1 (GLP-1) receptor agonists, which influence systemic and adipose tissue metabolism, may indirectly attenuate the persistence of inflammatory programming. In addition, targeting lipid metabolism and adipocyte–macrophage interactions may further destabilize the pro-inflammatory state. Importantly, sustained metabolic improvement, rather than transient correction, appears critical, as fluctuating metabolic conditions may reinforce rather than resolve inflammatory memory [2,42,43,44].

## 8. Targeting Memory Reactivation: Modulation of the Adipose Tissue Microenvironment

The adipose tissue microenvironment plays a decisive role in determining whether inflammatory memory remains latent or becomes reactivated. Local factors, including adipocyte-derived signals, extracellular matrix remodeling, hypoxia, and chronic low-grade inflammation, continuously shape ATM behavior. Therapeutic strategies at this level aim to modify the tissue context that drives recurrent activation of inflammatory pathways. Maintenance of weight stability, prevention of repeated cycles of weight loss and regain, and reduction in local inflammatory signaling may limit the reactivation of maladaptive immune responses. In addition, interventions targeting tissue hypoxia and fibrosis may further disrupt the microenvironmental cues that sustain inflammatory loops. These approaches emphasize that the adipose tissue niche is not merely a passive site of inflammation, but an active regulator of immune memory dynamics [45].

## 9. Challenges and Limitations in Targeting Inflammatory Memory

Despite its conceptual appeal, targeting inflammatory memory presents several challenges. First, the lack of specific biomarkers for identifying memory states in human adipose tissue limits patient stratification and therapeutic monitoring. Second, most mechanistic insights are derived from experimental models, and their translation to human physiology remains incomplete. Third, interventions aimed at modulating immune memory must balance efficacy with the risk of impairing host defense, particularly when targeting epigenetic or metabolic pathways with systemic effects. These limitations highlight the need for further studies integrating human data, longitudinal analyses, and tissue-specific approaches [46,47,48].

## 10. Future Perspectives

Targeting inflammatory memory in adipose tissue macrophages represents a shift from conventional anti-inflammatory strategies toward modulation of immune system programming. Rather than focusing on individual cytokines or signaling pathways, future therapeutic approaches should aim to disrupt the formation, persistence, or reactivation of inflammatory memory as an integrated process. Such strategies may be particularly relevant in clinical contexts characterized by repeated or chronic metabolic stress, including obesity, weight cycling, and metabolic recovery. A deeper understanding of these mechanisms may ultimately enable the development of interventions capable of reprogramming immune responses and reducing long-term cardiometabolic and oncologic risk.

## 11. The Obesity–Immunotherapy Paradox

Although obesity is a well-established risk factor for cancer development, accumulating evidence suggests that it may paradoxically enhance the efficacy of certain immunotherapies—a phenomenon referred to as the obesity–immunotherapy paradox.

Notably, PD-1 blockade has been shown to enhance macrophage glycolysis, increase the expression of CD86 and MHC class I/II molecules, and improve T-cell activation [36]. Interestingly, these effects appear to be context-dependent. In obese individuals, administration of leptin analogs has been associated with tumor progression, whereas PD-1 blockade leads to a reduction in tumor size and progression [49].

In contrast, in individuals with normal nutritional status, leptin administration appears to exert beneficial anti-tumor effects. Leptin enhances systemic inflammatory phenotypes, increasing CD86 and MHCII expression in macrophages. Experimental models have demonstrated that leptin-treated animals exhibit significantly smaller tumor volumes compared to controls, suggesting that elevated leptin levels may, under certain conditions, reduce overall tumor growth. Furthermore, leptin has been shown to partially reverse obesity-induced inhibitory effects on tumor-associated macrophages (TAMs), increasing MHCII expression and showing a trend toward higher CD86 expression, consistent with a shift toward a more M1-like phenotype [49].

This apparent paradox is most likely explained by leptin resistance in obesity, which alters downstream signaling and results in metabolic consequences similar to those observed in hyperinsulinemia, including activation of the PI3K/AKT signaling pathway.

These findings underscore the importance of metabolic context in shaping immune responses and highlight the need for personalized immunotherapeutic strategies in obese patients.

## 12. Discussion

The concept of inflammatory memory in adipose tissue macrophages (ATMs) provides a unifying framework that integrates prior inflammatory and metabolic exposures with future disease risk. In this review, we propose that inflammatory memory represents a persistent, context-dependent state of macrophage reprogramming, shaped by the interplay between epigenetic priming, metabolic reprogramming, and microenvironmental reinforcement. This integrative perspective helps to explain how transient or repeated metabolic insults can translate into long-lasting immune dysfunction and sustained low-grade inflammation.

A key implication of this framework is that inflammatory activation in adipose tissue should not be viewed as a purely reversible or stimulus-dependent process. Instead, macrophages may retain a “record” of prior exposures, resulting in a biased response to subsequent challenges. This is particularly relevant in clinical contexts such as obesity and weight cycling, where repeated fluctuations in metabolic status may reinforce maladaptive immune programming. In this sense, inflammatory memory may serve as a mechanistic link between past metabolic history and current disease phenotype, contributing to the heterogeneity observed among patients with similar anthropometric or biochemical profiles.

Importantly, while the concept of trained immunity has provided a foundation for understanding innate immune memory, its direct application to adipose tissue macrophages requires careful contextualization. Unlike circulating monocytes, ATMs reside within a complex and metabolically active microenvironment that continuously shapes their functional state. Adipocyte-derived signals, lipid flux, hypoxia, and extracellular matrix remodeling collectively influence macrophage behavior, suggesting that inflammatory memory in adipose tissue is not solely a cell-intrinsic phenomenon but rather a dynamically regulated process. This distinction may explain why findings from experimental models do not always fully translate to human physiology and highlights the importance of tissue-specific investigation.

Another critical aspect is the interaction between epigenetic and metabolic mechanisms in sustaining inflammatory memory. Epigenetic priming provides a structural basis for rapid transcriptional activation, while metabolic reprogramming ensures the energetic and biosynthetic support required for maintaining this state. These processes are further modulated by the adipose tissue niche, creating a self-reinforcing loop that stabilizes inflammatory phenotypes over time. Disruption of this loop may therefore represent a key therapeutic objective.

Despite the conceptual advances outlined in this review, several limitations must be acknowledged. First, direct evidence of inflammatory memory in human adipose tissue macrophages remains limited, with much of the current understanding derived from experimental models and indirect observations. Second, the lack of standardized markers for identifying memory states in tissue-resident macrophages poses a challenge for both research and clinical translation. Third, the temporal dynamics of inflammatory memory—particularly its duration, reversibility, and responsiveness to intervention—remain incompletely understood.

From a clinical perspective, the recognition of inflammatory memory has important implications for disease prevention and management. Strategies that focus solely on short-term metabolic correction may be insufficient if underlying immune programming persists. This may partly explain why some patients fail to achieve sustained benefit despite apparent metabolic improvement. Conversely, interventions that promote long-term metabolic stability and reduce repeated inflammatory stimuli may have a more profound impact by preventing the reinforcement of maladaptive memory states. In this context, weight stability, rather than repeated cycles of loss and regain, may represent an underappreciated therapeutic goal.

Future research should aim to bridge the gap between experimental findings and human disease by integrating longitudinal clinical studies, tissue-specific analyses, and advanced molecular profiling. Particular attention should be given to identifying biomarkers of inflammatory memory, characterizing ATM heterogeneity, and elucidating the reversibility of memory states under different therapeutic conditions. Such efforts may ultimately enable the development of targeted interventions capable of reprogramming macrophage function and reducing long-term cardiometabolic and oncologic risk.

## 13. Conclusions

In conclusion, inflammatory memory in adipose tissue macrophages represents a conceptual shift in our understanding of chronic inflammation in metabolic disease. By linking past exposures to future outcomes through integrated cellular mechanisms, this framework offers new insights into disease pathogenesis and opens avenues for more effective and durable therapeutic strategies.

## Figures and Tables

**Table 1:** **Mechanistic framework of inflammatory memory in adipose tissue macrophages.** Metabolic and inflammatory stimuli, including obesity, excess nutrients, hypoxia, and weight cycling, induce persistent functional reprogramming in adipose tissue macrophages. This process is mediated through three interconnected mechanisms: (1) epigenetic priming, which encodes prior exposures via chromatin remodeling; (2) metabolic reprogramming, which sustains the inflammatory phenotype through altered cellular metabolism; and (3) microenvironmental reinforcement, in which adipose tissue-specific signals continuously modulate and reactivate macrophage responses. These self-reinforcing processes collectively link past exposures to future pathological outcomes, including insulin resistance, atherosclerosis, and cancer progression.

Mechanism	Key Features	Human Evidence	Experimental Evidence (Animal/In Vitro)	Strength of Evidence
Epigenetic priming [12,13]	Persistent chromatin remodeling (H3K4me3, H3K27ac), enhancer activation, transcriptional memory	Limited direct evidence in adipose tissue macrophages; indirect support from circulating monocytes in obesity and metabolic syndrome	Strong evidence from trained immunity models; macrophages show stable epigenetic changes after metabolic/inflammatory stimuli	Moderate (indirect human, strong experimental)
Metabolic reprogramming [14,15]	Shift toward glycolysis, altered mitochondrial function, lipid handling, metabolite signaling (e.g., succinate, acetyl-CoA)	Evidence of altered metabolic profiles in adipose tissue macrophages in obesity; human adipose biopsies show metabolic–inflammatory coupling	Robust evidence in murine and in vitro macrophage models demonstrating sustained metabolic rewiring linked to inflammatory activation	Moderate–strong
Microenvironmental reinforcement [16,17]	Adipocyte–macrophage crosstalk, cytokine loops, extracellular matrix remodeling, hypoxia	Strong evidence from human adipose tissue studies showing local inflammatory circuits and ATM heterogeneity in obesity	Extensive evidence from animal models confirming niche-driven macrophage activation and persistence	Strong
Persistence of activation [18]	Sustained inflammatory phenotype after removal of initial stimulus	Limited direct human longitudinal data; indirect evidence from chronic low-grade inflammation in obesity	Demonstrated in trained immunity and metabolic memory models; macrophages retain altered responsiveness over time	Moderate
Reactivation upon secondary stimulus [19,20]	Exaggerated response to subsequent inflammatory/metabolic challenge	Sparse direct human evidence; inferred from clinical worsening with repeated metabolic insults (e.g., weight cycling)	Strong experimental evidence showing amplified cytokine response upon re-stimulation	Moderate (conceptual + experimental)
Clinical correlates [2,21,22,23]	Association with insulin resistance, atherosclerosis, tumor progression	Strong epidemiological and clinical associations between adipose inflammation and cardiometabolic/cancer risk	Mechanistic support from preclinical models linking macrophage activation to disease progression	Strong (association), moderate (causality)

## Data Availability

Data sharing is not applicable as no new data were generated or analyzed in this study.

## Abbreviations

The following abbreviations are used in this manuscript:

AKT	protein kinase B (PKB)
AMP	adenosine monophosphate
ACE2	angiotensin-converting enzyme 2
ASH1L	ASH1-like histone lysine methyltransferase
ASIC1	acid-sensing ion channel subunit 1
ATF6	activating transcription factor 6
ATG9A	autophagy related 9A
ATGL	adipose triglyceride lipase
BMI	body mass index
BRCA	breast cancer gene
bZIP	basic leucine zipper
C/EBPα	CCAAT/enhancer-binding protein alpha
cAMP	cyclic adenosine monophosphate
CBP	CREB-binding protein
CCL18	CC motif chemokine ligand 18
CD4+ T	cluster of differentiation 4-positive T cells
CD8+ T	cluster of differentiation 8-positive T cells
CD9	tetraspanin-29
CD68^+^	cluster of differentiation 68 (macrosialin)
CD163+	cluster of differentiation 163, M2-type tumor-associated macrophage marker
CLS	crown-like structures
CREB	cyclic AMP-responsive element-binding protein
CRTC2	CREB-regulated transcription coactivator 2
CRTC3	CREB-regulated transcription coactivator 3
ECATG9a	ATG9A analog
eIF4E	eukaryotic translation initiation factor 4E
ER	endoplasmic reticulum
ERK1/2	extracellular signal-regulated kinases 1/2
ETV4	ETS variant transcription factor 4
FSP1	ferroptosis suppressor protein 1
FOXO1	forkhead box protein O1
GLP-1	glucagon-like peptide-1
GPCRs	G protein–coupled receptors
H3K4me3	histone H3 lysine 4 trimethylation
HER2	human epidermal growth factor receptor 2
HSL	hormone-sensitive lipase
IFN-γ	interferon-gamma
IGF-1	insulin-like growth factor 1
IHC	immunohistochemistry
IL-1β	interleukin-1 beta
IL-4	interleukin-4
IL-6	interleukin-6
IL-13	interleukin-13
IRE1	inositol-requiring enzyme 1
ISG15	interferon-stimulated gene 15
ISG56	interferon-stimulated gene 56
JAK	Janus kinase
JNK	c-Jun N-terminal kinase
KID	kinase-inducible domain
KLF10	Krüppel-like factor 10
LDL	low-density lipoprotein
LIPA	lipase A
LPL	lipoprotein lipase
LPS	lipopolysaccharide
MAPK	mitogen-activated protein kinase
MASH	metabolic dysfunction-associated steatohepatitis
MHC	major histocompatibility complex
MHCII	major histocompatibility complex class II
miR-29b	microRNA-29b
miR-149	microRNA-149
miR-191	microRNA-191
MLKL	mixed lineage kinase domain-like pseudokinase
MMPs	matrix metalloproteinases
MMP9	matrix metalloproteinase-9
mTOR	mechanistic target of rapamycin
mTORC1	mechanistic target of rapamycin complex 1
NF-κB	nuclear factor kappa-light-chain-enhancer of activated B cells
NK	natural killer cells
NR3C1	nuclear receptor subfamily 3 group C member 1
PAAu BPs	adipose-targeting and apoptotic cell-mimicking gold nanobipyramids
p16INK4a	cyclin-dependent kinase inhibitor 2A
p38	p38 mitogen-activated protein kinase
PD-1	programmed cell death protein 1
PERK	protein kinase RNA-like endoplasmic reticulum kinase
PI3K	phosphoinositide 3-kinase
PIP3	phosphatidylinositol-3,4,5-trisphosphate
PKR	protein kinase R (eukaryotic translation initiation factor 2-alpha kinase 2)
PPAR-γ	peroxisome proliferator-activated receptor gamma
RTKs	receptor tyrosine kinases
RIPK1	receptor-interacting protein kinase 1
RIPK3	receptor-interacting protein kinase 3
RVI	regulatory volume increase
SIK2	salt-inducible kinase 2
SMAD	mothers against decapentaplegic homolog
solTNFα	soluble tumor necrosis factor-alpha
STAT	signal transducer and activator of transcription
SGLT-2	sodium-glucose co-transporter 2
TBARS	thiobarbituric acid reactive substances
TAM	tumor-associated macrophage
TGF-β1	transforming growth factor-beta 1
TIEG1	TGFβ-inducible early gene 1
TME	tumor microenvironment
TMPRSS2	transmembrane serine protease 2
TNFα	tumor necrosis factor-alpha
TNFR1	tumor necrosis factor receptor type 1
TNFR2	tumor necrosis factor receptor type 2
TREM2	triggering receptor expressed on myeloid cells 2
UPR	unfolded protein response
VCAM-1	vascular cell adhesion molecule-1
VEGF	vascular endothelial growth factor
VLDL	very low-density lipoproteins
Wnt	wingless-related integration site
ZBP1	Z-form nucleic acid-binding protein 1
3T3-L1	3T3 Swiss albino mouse embryonal fibroblasts
8-PGF2α	8-iso-prostaglandin F2α

## References

[1] Hagberg C.E., Spalding K.L. (2024). White adipocyte dysfunction and obesity-associated pathologies in humans. Nat. Rev. Mol. Cell Biol..

[2] Tomić Naglić D., Mandić A., Milankov A., Pejaković S., Janičić S., Vuković N., Bajkin I., Ičin T., Manojlović M., Stokić E. (2024). Metabolic dysregulation in obese women and the carcinogenesis of gynecological tumors: A review. Biomol. Biomed..

[3] Dalamaga M., Liu J. (2022). A chromatin remodeling checkpoint of diet-induced macrophage activation in adipose tissue. Metab. Open.

[4] Jang I.H., Carey A., Kruglov V., Nguyen K., Misialek J.R., Cholensky S.H., Smith D.M., Bai S., Nottoli T., Bernlohr D.A. (2026). GDF3 promotes adipose tissue macrophagemediated inflammation via altered chromatin accessibility during aging. Nat. Aging.

[5] Li W., Wei J., Li L., Sun W. (2026). Trained immunity in neutrophils and mononuclear phagocytes: Mechanisms and pathophysiological functions. Cells.

[6] Sun S., Barreiro L.B. (2020). The epigenetically-encoded memory of the innate immune system. Curr. Opin. Immunol..

[7] Cordani M., Rumio C., Bontempi G., Strippoli R., Marcucci F. (2025). Oxidative and glycolytic metabolism: Their reciprocal regulation and dysregulation in cancer. Cells.

[8] Richhariya S., Shin D., Schlichting M., Rosbash M. (2025). Metabolic rewiring prevents neurodegeneration caused by chronic mitochondrial dysfunction. Curr. Biol..

[9] Raynor J.L., Chi H. (2026). Immunometabolic and spatiotemporal control of tissue-resident memory T cell biology. Barrier Immun..

[10] Cui Y., Feng Z., Zhao Q., Dai H., Zheng Y., Rui H., Liu B. (2025). Immunocyte lipid metabolic reprogramming: A novel pathway for targeted intervention in autoimmune diseases. Front. Immunol..

[11] Liu Y., Huang X., Sang L., Zhang Y., Cao J., Kong Q. (2026). Modulation and reprogramming of adipose tissue macrophages in obesity. Biomolecules.

[12] Pham T.H., Benner C., Lichtinger M., Schwarzfischer L., Hu Y., Andreesen R., Chen W., Rehli M. (2012). Dynamic epigenetic enhancer signatures reveal key transcription factors associated with monocytic differentiation states. Blood.

[13] Pei J., Harakalova M., Treibel T.A., Lumbers R.T., Boukens B.J., Efimov I.R., van Dinter J.T., González A., López B., El Azzouzi H. (2020). H3K27ac acetylome signatures reveal the epigenomic reorganization in remodeled non-failing human hearts. Clin. Epigenet..

[14] Kishida T., Ejima A., Yamamoto K., Tanaka S., Yamamoto T., Mazda O. (2015). Reprogrammed functional brown adipocytes ameliorate insulin resistance and dyslipidemia in diet-induced obesity and type 2 diabetes. Stem Cell Rep..

[15] Seim G.L., Fan J. (2022). A matter of time: Temporal structure and functional relevance of macrophage metabolic rewiring. Trends Endocrinol. Metab..

[16] Sárvári A.K., Doan-Xuan Q.M., Bacsó Z., Csomós I., Balajthy Z., Fésüs L. (2015). Interaction of differentiated human adipocytes with macrophages leads to trogocytosis and selective IL-6 secretion. Cell Death Dis..

[17] Jiménez-Cortegana C., Gutiérrez-García C., Sánchez-Jiménez F., Vilariño-García T., Flores-Campos R., Pérez-Pérez A., Garnacho C., Sánchez-León M.L., García-Domínguez D.J., Hontecillas-Prieto L. (2024). Impact of obesity-associated myeloid-derived suppressor cells on cancer risk and progression. Int. J. Oncol..

[18] Kardinal R., Wachten D. (2025). Macrophages in metaflammation—Fueling chronic inflammation in metabolic disease. Pflug. Arch..

[19] Cottam M.A., Itani H.A., Beasley A.A., Hasty A.H. (2018). Links between immunologic memory and metabolic cycling. J. Immunol..

[20] Kazmierczak B., Volpert V. (2025). Mathematical modelling of tissue growth control by positive and negative feedbacks. PLoS ONE.

[21] Turner L., Wanasinghe A.I., Brunori P., Santosa S. (2025). Is adipose tissue inflammation the culprit of obesity-associated comorbidities?. Obes. Rev..

[22] De Fano M., Bartolini D., Tortoioli C., Vermigli C., Malara M., Galli F., Murdolo G. (2022). Adipose tissue plasticity in response to pathophysiological cues. Int. J. Mol. Sci..

[23] Patel P., Abate N. (2013). Role of subcutaneous adipose tissue in the pathogenesis of insulin resistance. J. Obes..

[24] Zhou F., Li X., Jiang J., Zhang L. (2026). Understanding obesity–cancer crosstalk. Mater. Today Bio.

[25] Moukarzel L.A., Ferrando L., Stylianou A., Lobaugh S., Wu M., Nobre S.P., Iasonos A., Zoppoli G., Giri D.D., Abu-Rustum N.R. (2022). Impact of obesity and adipose tissue inflammation on the omental microenvironment in endometrial cancer. Cancer.

[26] Sun K., Tordjman J., Clément K., Scherer P.E. (2013). Fibrosis and adipose tissue dysfunction. Cell Metab..

[27] Li F., Sun H., Li Y., Bai X., Dong X., Zhao N., Meng J., Sun B., Zhang D. (2021). High expression of eIF4E is associated with tumor macrophage infiltration. BMC Cancer.

[28] Miranda A.M.A., McAllan L., Mazzei G., Andrew I., Davies I., Ertugrul M., Kenkre J., Kudo H., Carrelha J., Patel B. (2025). Selective remodelling of the adipose niche in obesity and weight loss. Nature.

[29] Galli G., Saleh M. (2021). Immunometabolism of macrophages in bacterial infections. Front. Cell Infect. Microbiol..

[30] Caceres L., Guha Ray A., Emont M.P., Weinstock A. (2025). Influence of weight loss and weight regain on adipose tissue inflammation. Arterioscler. Thromb. Vasc. Biol..

[31] Russo S., Kwiatkowski M., Govorukhina N., Bischoff R., Melgert B.N. (2021). Meta-inflammation and metabolic reprogramming of macrophages in diabetes and obesity. Front. Immunol..

[32] Wu T., Shao Y., Li X., Wu T., Yu L., Liang J., Zhang Y., Wang J., Sun T., Zhu Y. (2023). NR3C1 activation promotes pancreatic β-cell autophagy overload. Autophagy.

[33] Esguerra J.L.S., Ofori J.K., Nagao M., Shuto Y., Karagiannopoulos A., Fadista J., Sugihara H., Groop L., Eliasson L. (2020). Glucocorticoid induces human beta cell dysfunction by involving riborepressor GAS5 LincRNA. Mol. Metab..

[34] Calle E.E., Rodriguez C., Walker-Thurmond K., Thun M.J. (2003). Overweight, obesity, and mortality from cancer in a prospectively studied cohort of U.S. adults. N. Engl. J. Med..

[35] Bader J.E., Wolf M.M., Lupica-Tondo G.L., Madden M.Z., Reinfeld B.I., Arner E.N., Hathaway E.S., Steiner K.K., Needle G.A., Hatem Z. (2024). Obesity induces PD-1 on macrophages to suppress anti-tumour immunity. Nature.

[36] Monteiro J.F., Fernandes A., Tato D.G., Moreira E., Ribeiro R., Reguengo H., Gonçalves J., Fresco P. (2025). Optimizing anti-PD1 immunotherapy. Cancers.

[37] Hinte L.C., Castellano-Castillo D., Ghosh A., Melrose K., Gasser E., Noé F., Massier L., Dong H., Sun W., Hoffmann A. (2024). Adipose tissue retains an epigenetic memory of obesity after weight loss. Nature.

[38] Ussar S. (2025). Targeting adipose inflammation after weight loss. Diabetes.

[39] Zovkic I.B., Guzman-Karlsson M.C., Sweatt J.D. (2013). Epigenetic regulation of memory formation. Learn. Mem..

[40] Song J., Yang P., Chen C., Ding W., Tillement O., Bai H., Zhang S. (2025). Targeting epigenetic regulators in cancer therapy resistance. Signal Transduct. Target. Ther..

[41] Tarulli I., Toscano-Rivalta R., Watt L., Gräff J. (2025). Epigenetic mechanisms of memory allocation. J. Neurochem..

[42] Yang L., Zhang C., Zhang Z., Yao S., Shen J., Zhang Z., Xiao Z., Wang S., Wu Z. (2026). Targeting metabolic reprogramming to enhance immunotherapy. J. Transl. Med..

[43] Cui W., Lv C., Geng P., Fu M., Zhou W., Xiong M., Li T. (2024). Metformin in dementia: New insights. Front. Pharmacol..

[44] Kumar V. (2025). GLP-1/GLP-1R axis from metabolism to immunity. Open Biol..

[45] Lo Iacono M., Modica C., Porcelli G., Brancato O.R., Muratore G., Bianca P., Gaggianesi M., Turdo A., Veschi V., Todaro M. (2022). Targeting adipose tissue microenvironment. Biomolecules.

[46] Vo M.C., Tran V.D., Nguyen V.T., Ruzimurodov N., Trung D.T., Kim S.K., Jung S.-H., Lee J.J. (2025). CAR-T therapy in solid tumors. J. Hematol. Oncol..

[47] Schlüter T., van Elsas Y., Priem B., Ziogas A., Netea M.G. (2025). Trained immunity: Inflammatory memory in disease. Cell Res..

[48] Xu W., Guo Z., Xu T., Chen J., Chen L., Xu W. (2025). Reversing inflammatory diseases via trained immunity. Front. Immunol..

[49] Dudzinski S.O., Bader J.E., Beckermann K.E., Young K.L., Hongo R., Madden M.Z., Abraham A., Reinfeld B.I., Ye X., MacIver N.J. (2021). Leptin augments antitumor immunity. J. Immunol..

